# Preoperative inflammatory and molecular marker changes in 125 supratentorial glioma cases

**DOI:** 10.55730/1300-0144.6143

**Published:** 2025-11-06

**Authors:** Semih Can ÇETİNTAŞ, Burak TAHMAZOĞLU, Tufan Agah KARTUM, Rahşan KEMERDERE, Taner TANRIVERDİ

**Affiliations:** Department of Neurosurgery, Cerrahpaşa Medical Faculty, İstanbul University-Cerrahpaşa, İstanbul, Turkiye

**Keywords:** Gliomas, 1p/19q codeletion, Ki-67 labeling index, IDH1 mutation, preoperative systemic inflammatory marker

## Abstract

**Background/aim:**

To determine whether there is a possible link between selected preoperative systemic inflammatory markers and molecular pathological indices.

**Material and methods:**

For this retrospective study, 125 patients served as subjects. Preoperative systemic inflammatory markers, namely neutrophil to lymphocyte ratio (NLR), platelet to lymphocyte ratio (PLR), and lymphocyte to monocyte ratio (LMR), and postoperative pathological indices, namely mitosis, Ki-67 labeling index (LI), isocitrate dehydrogenase-1 (IDH1) mutation, and 1p/19q codeletion, were obtained from the electronic database.

**Results:**

Results showed that NLR positively correlated with PLR (p = 0.00001), mitotic activity (p = 0.001), and Ki-67 LI (p = 0.00001). On the other hand, LMR *negatively* correlated with PLR (p = 0.00001), mitotic activity (p = 0.00001), Ki-67 LI (p = 0.001), and IDH1 mutation (p = 0.04).

**Conclusion:**

Our results suggest a link between systemic inflammation and histopathology, and higher NLR and/or lower LMR may be a novel prognostic marker in gliomas. A prospective multicenter study with a larger patient population would be more convincing. Until such a study is published, the results of retrospective studies, including ours, should be evaluated with caution.

## 1. Introduction

Gliomas are the most common primary brain tumors, accounting for almost 80% of all malignant brain tumors [1]. Despite multimodal treatments including surgery, chemotherapy, radiotherapy, immunotherapy, and even targeted therapy, the recurrence rate is very high, especially in grade-IV gliomas or glioblastoma multiforme (GBM), in which the median survival is around 15 months. The main reason for the high recurrence rate is its invasive nature, and total surgical removal of gliomas is challenging. Searching for novel molecular markers to predict prognosis and histological grade has become popular. Thus, several molecular markers, which are thought to play an important role in the development and/or progression of gliomas, have been identified [2,3]. However, a few are valuable enough to be used in clinical practice. There is a common notion in the literature that identifying a novel molecular marker may enable clinicians to predict prognosis and provide effective management in the future.

One such marker is isocitrate dehydrogenase 1/2 (IDH), a Krebs cycle enzyme that generates glutathione, a major antioxidant, in cells [4]. The presence of IDH mutation was primarily found in low-grade gliomas (LGGs) and secondary high-grade gliomas (HGGs) [5]. Previous studies have shown that IDH-mutant gliomas have a significantly better overall survival (OS) compared with wild-type (IDH-nonmutant) IDH gliomas. Furthermore, IDH1 mutations are associated with better progression-free survival (PFS) [6,7].

The nonhistone protein Ki-67 is expressed in proliferating cells and has been used to distinguish growing from nongrowing cells [8]. An accumulating body of evidence has shown that Ki-67 is a reliable marker of cell proliferation, and its overexpression is associated with worse prognosis, shorter OS, and higher glioma grade [9,10].

Inflammation is the hallmark of cancer, and the increasing body of evidence has shown that tumor progression is related not only to the tumor itself but also to the systemic inflammation [11]. Recent research has focused on systemic inflammatory markers for the prognosis prediction and histological grade. Lately, there has been a common notion that preoperative systemic inflammatory markers, especially the neutrophil-to-lymphocyte ratio (NLR), platelet-to-lymphocyte ratio (PLR), and lymphocyte-to-monocyte ratio (LMR), may be prognostic markers in glioma [2,12]. NLR and PLR have been shown to be independent prognostic factors for OS, and low NLR is associated with a good prognosis [12]. In addition, low LMR (less likely PLR) could serve as an index for distinguishing GBM from brain metastasis [13,14]. Furthermore, higher NLR and PLR were correlated with glioma grade, and NLR showed the highest accuracy for predicting glioma grade [15]. However, almost all previous studies are retrospective, and the results need to be confirmed by future prospective clinical trials.

The aim of this retrospective study is to determine whether there is a possible association between selected preoperative systemic inflammatory markers and molecular pathological indices, including mitosis, Ki-67 labeling index (LI), IDH1 mutation, and 1p/19q codeletion. Furthermore, we aimed to see whether there is a difference regarding the parameters between gender (male vs. female), side (right vs. left), lobes involved (lobar vs. multilobar), grade (LGG vs. HGG), histologic type (GBM vs. non-GBM), mitosis (present vs. absent), IDH1 mutation (mutant vs. nonmutant), 1p/19q codeletion (deleted vs. nondeleted), and World Health Organization (WHO) subgrades (grade I through IV).

### 1.1. Patient collection

Our local ethics committee approved this study, and all invasive procedures were performed after each patient signed informed consent. The authors of this study declare that some patient data were used in our previous studies [13–15]. The present study included the patients who underwent surgery on supratentorial glioma between 2010 and 2024. A single surgeon performed all the surgeries. We reviewed the electronic medical records and collected the following data: age, sex, location, side, pathologic diagnosis, including the 2016 WHO grade [16], neutrophil, lymphocyte, platelet, and monocyte counts, NLR, PLR, LMR, mitosis, Ki-67 LI, IDH1 mutation, and 1p/19q codeletion. Thus, between 2010 and 2024, 287 patients with supratentorial gliomas underwent surgery, and after applying the selection criteria, 125 were included in this study. Our inclusion criteria were as follows: patients who were 1) older than 18 years old; had 2) a histologically confirmed glioma; 3) no chemotherapy and/or radiotherapy prior to surgery; 4) no other systemic infection or organ diseases, and 5) a complete blood test. In this study, low-grade glioma (LGG) and high-grade glioma (HGG) were defined as grades I and II, and grades III and IV, respectively.

## 2. Methods

### 2.1. Laboratory tests

We collected peripheral venous blood for routine preoperative work-up, and neutrophil (10^3^/mm^3^), lymphocyte (10^3^/mm^3^), monocyte (10^3^/mm^3^), and platelet (10^3^/mm^3^) counts were recorded. The preoperative NLR (the quotient of the absolute number of neutrophil count to lymphocyte count), PLR (the quotient of the absolute number of platelet to lymphocyte count), and LMR (the quotient of the absolute number of lymphocyte to monocyte count) were calculated.

### 2.2. Molecular pathological indices

Molecular pathological indices, including mitosis, Ki-67 LI, IDH1 mutation, and 1p/19q codeletion, were reviewed from the pathology reports. The number of mitoses was described as mitosis/10 high-power fields (10/HPF), and Ki-LI was expressed as a percentage.

### 2.3. Statistical analysis

Statistical analysis was performed using SPSS version 22.0. Frequencies and percentages were used for categorical variables, and the chi-square test was used for group comparisons. A one-sample Kolmogorov–Smirnov test was used for normality. Normally distributed data were expressed as mean ± standard deviation, and nonnormally distributed data were expressed as median and interquartile range (25^th^ to 75^th^ percentiles). Comparisons between normally distributed data were evaluated by the independent samples t-test, while nonnormally distributed data were evaluated by the nonparametric Mann–Whitney U test. Multiple (more than two) group comparisons of normally distributed data were evaluated by one-way analysis of variance (ANOVA) with an appropriate post hoc test, and nonnormally distributed data were evaluated by the nonparametric Kruskal–Wallis test. Spearman correlation tests were used for normal and nonnormal distributed data, respectively. P-values less than 0.05 (p < 0.05) were considered to be statistically significant.

## 3. Results

This study included 125 patients with a mean age of 41.80 ± 13.81 years, who underwent surgery on supratentorial glioma. Of the 125 patients, 66 (52.8%) had left-sided gliomas and 59 (47.2%) had right-sided gliomas. The side preference was toward the left, but the difference was not significant (chi-square test; p = 0.53). Every lobe was involved, but the most common locations were the temporal (n = 42; 33.6%) and frontal (n = 37; 29.6%) lobes. The most common pathological diagnosis in this series was grade II (n = 53; 42.4%), followed by grade IV (n = 38; 30.4%), grade III (n = 21; 16.8%), and grade I (n = 13; 10.4%). Normal-distributed data included lymphocyte and platelet counts and LMR, with mean values of 1.96 ± 0.88, 246.80 ± 69.57, and 3.78 ± 1.84, respectively. In this series, nonnormal distributed data included neutrophil and monocyte counts, NLR, PLR mitosis, and Ki-67 LI with median values of 2.67, 0.50, 2.67, 135.62, 3, and 6, respectively. With respect to IDH1 positivity (mutant) status, 71 gliomas (56.8%) were mutant, while 47 gliomas (37.6%) were nonmutant. IDH1 mutation was not evaluated in 7 gliomas (5.6%) in this series. Codeletion of 1p and 19q was present in 26 (20.8%) gliomas and absent in 24 (19.2%); in 75 gliomas (60%), codeletion was not evaluated.

We wanted to show whether there was a correlation between selected preoperative hemogram parameters and molecular pathological indices. Spearman’s correlation analysis showed a weak positive correlation between neutrophil and platelet counts (r = 0.17; p = 0.04). In addition, neutrophil counts moderately correlated with monocyte count (r = 0.41; p = 0.00001), mitosis (r = 0.31; p = 0.00001), and Ki-67 LI (r = 0.34; p = 0.00001). Lymphocyte count showed a weak and moderate correlation with platelet (r = 0.27; p = 0.02) and monocyte counts (r = 0.31; p = 0.00001), respectively. A weak correlation between monocyte count and mitosis (r = 0.23; p = 0.008) and Ki-67 LI (r = 0.22; p = 0.01) was found. As expected, a robust positive correlation was observed between mitosis and Ki-67 LI (r = 0.88; p = 0.00001).

We further wondered whether there was an association between selected preoperative systemic inflammatory markers and molecular pathological indices. Spearman correlation analysis showed moderate correlations between NLR and PLR (r = 0.56; p = 0.00001), NLR and mitosis (r = 0.28; p = 0.001), and NLR and Ki-67 LI (r = 0.31; p = 0.00001). A moderate *negative* correlation was found between NLR and LMR (r = −0.63; p = 0.00001). Likewise, PLR showed a *negative*, moderate correlation with LMR (r = −0.47; p = 0.00001). A moderate *negative* correlation was noted between LMR and mitosis (r = −0.31; p = 0.00001), LMR and Ki-67 LI (r = −0.30; p = 0.001). Categorical comparisons (present/absent) showed that IDH1 mutation was moderately positively correlated with 1p/19q codeletion (r = 0.46; p = 0.00001) and weakly negatively correlated with LMR (r = −0.17; p = 0.04).

### 3.1. Differences between the genders

Of the 125 patients, 75 (60%) were male and 50 (40%) were female; the sex distribution was significantly different (chi-square test; p = 0.02). Females had significantly higher platelet counts than males (p = 0.01), but males showed significantly higher monocyte counts (p = 0.04), mitotic activity (p = 0.006), and Ki-67 LI (p = 0.009). Table 1 shows the statistical summary between the two genders.

### 3.2. Differences between the sides

In this series, 66 (52.8%) and 59 (47.2%) gliomas were located in the left and right sides, respectively, without any significant difference (chi-square test; p = 0.53). Expectedly, no parameters studied showed significant differences between the two sides (Table 2).

### 3.3. Differences between the numbers of lobes involved

Lobar and multilobar involvements were noted in 99 (79.2%) and 26 (20.8%) patients, respectively, and the difference was significant (chi-square test; p = 0.00001). Although the number of patients with multilobar involvement was fewer than those with lobar involvement, multilobar gliomas showed significantly higher neutrophil counts (p = 0.007), NLR (p = 0.007), mitotic activity (p = 0.02), and Ki-67 LI (p = 0.04). On the other hand, lymphocyte count (p = 0.01) and LMR (p = 0.02) were significantly higher in lobar involvement (Table 3).

### 3.4. Differences between the grades (low grade vs. high grade)

Low- and high-grade gliomas in this series comprised 67 (53.6%) and 58 (46.4%) patients, respectively, without a significant difference (chi-square test; p = 0.42). Neutrophil (p = 0.003) and monocyte counts (p = 0.01) and NLR (p = 0.01) were significantly higher in HGG, but LMR was significantly higher in LGG (p = 0.004). As expected, HGG showed higher levels of mitotic activity and Ki-67 LI than LGG, and the differences were statistically significant (p = 0.00001 for each parameter) (Table 4).

### 3.5. Differences between the GBM and non-GBM

Histopathological diagnosis in this report was GBM in 38 (30.4%) patients and non-GBM in 87 (69.6%) patients; the difference was statistically significant in favor of the non-GBM group (chi-square test; p = 0.00001). Patients with GBM showed significantly higher neutrophil counts (p = 0.00001) and NLR (p = 0.00001), but LMR was significantly higher in the non-GBM group (p = 0.01). Although the GBM group was half the size of the non-GBM group, mitotic activity and Ki-67 LI showed a strong, significant difference in the GBM group (p = 0.00001 for each parameter). Table 5 shows a statistical summary.

### 3.6. Differences between the presence and absence of mitosis

Mitoses were noted in 94 (75.2%) gliomas, and the difference was significant compared with gliomas without mitosis (chi-square test; p = 0.00001). Neutrophil (p = 0.01) and monocyte (p = 0.01) counts and NLR (p = 0.03) were significantly higher in gliomas with mitosis. As expected, Ki-67 LI (p = 0.00001) showed a significant difference between gliomas with mitosis. Here, LMR is worth mentioning. Although the number of gliomas without mitosis was one-third of the number of gliomas with mitosis, LMR was higher in gliomas without mitosis, and the difference tended to be significant (p = 0.06) (Table 6).

### 3.7. Differences between the mutant and nonmutant IDH1 gliomas

Mutant and nonmutant IDH1 were detected in 71 (56.8%) and 47 (37.6%) gliomas, and the difference was significant (chi-square test; p = 0.00001). Among systemic preoperative inflammatory markers, only LMR was significantly elevated in IDH1-mutant gliomas (p = 0.04). As expected, mean mitotic activity (p = 0.00001) and Ki-67 LI (p = 0.00001) were significantly higher in IDH1 nonmutant gliomas (Table 7).

### 3.8. Differences between the presence and absence of 1p/19q codeletion

In this series, 1p/19q codeletion was positive in 26 (20.8%) gliomas and negative in 24 (19.2%). No significant difference was noted (chi-square test; p = 0.77) (Table 8). None of the preoperative systemic inflammatory markers showed significant differences. However, mean mitotic activity (p = 0.02) and Ki-67 LI (p = 0.04) were significantly higher in gliomas without 1p/19q codeletion.

### 3.9. Differences between the WHO glioma grades

The diagnosis of gliomas in this series was based on histopathology reports: grade I (10.4%), II (42.4%), III (16.8%), and IV (30.4%). The difference among the grades was significant (p = 0.00001). One-way ANOVA with post hoc Hochberg’s test was applied for normally distributed data (lymphocyte and platelet counts, and LMR), and nonparametric Kruskal–Wallis test was used for the rest (for nonnormally distributed data) for multiple comparisons. Multiple group comparisons showed significant differences in neutrophil (p = 0.00001) and monocyte (p = 0.02) counts, NLR (p = 0.00001), and Ki-67 LI (p = 0.00001). Table 9 summarizes the statistical comparisons among the WHO grades of gliomas.

Post hoc Hochberg’s test showed that LMR was significantly higher in grade-II glioma than in grade-IV glioma (p = 0.02).

For nonnormally distributed data, nonparametric tests showed that neutrophil count was significantly higher in grade-IV gliomas compared with grade I (p = 0.00001) and grade II (p = 0.00001). No significant difference was noted between grade-III and grade-IV gliomas. Grade-III and grade-IV gliomas had significantly higher neutrophil counts than grade-II gliomas (p = 0.02 and 0.01, respectively).

NLR in grade-IV was significantly higher compared with grade-I (p = 0.001), grade-II (p = 0.003), and grade-III gliomas (p = 0.002).

Mitotic activity was highest in grade-IV gliomas, followed by grade-III gliomas. Significantly higher levels of median mitotic activity were noted in grade IV when compared with grade-I (p = 0.00001), grade-II (p = 0.00001), and grade-III gliomas (p = 0.003). Furthermore, a significantly higher level was observed in grade-III gliomas compared with grade-I (p = 0.00001) and grade-II (p = 0.00001). No difference was noted between grade-I and II gliomas.

Ki-67 LI was highest in grade IV gliomas, followed by grade-III gliomas. Significantly higher levels were found in grades II to IV gliomas when compared with grade-I gliomas (p = 0.00001 for each). Again, grade-IV showed significantly higher levels than grade-II (p = 0.00001) and grade-III gliomas (p = 0.00001). Additionally, grade-III gliomas had a higher level compared with grade-II gliomas (p = 0.00001).

## 4. Discussion

Neutrophils, lymphocytes, monocytes, and platelets are important mediators in both local and systemic inflammatory responses to tumors. It is well known that neutrophils are the most abundant leukocytes and are the first to be recruited to inflammation sites [17]. Tumor cells, by secreting reactive oxygen species, increase the number of neutrophils both within the microenvironment around and inside the tumor itself [17]. As a result, the blood–brain barrier is impaired, allowing more monocytes to reach the inflammatory sites. Previously published studies indicated that increased neutrophil counts are associated with glioma grade [15,18]. Lymphocytes exert antitumor effects by inhibiting tumor cell proliferation [19]. On the other hand, monocytes differentiate into tumor-associated macrophages, which promote tumor growth [20]. The interaction between platelets and tumor cells is complex and not well understood. The primary function of platelets is to repair damaged tissue. In chronic inflammatory conditions, such as tumors, platelets stimulate tumor growth and angiogenesis through secreted proinflammatory mediators [21]. The results of previous studies reporting preoperative hematological markers in glioma and other brain tumors are consistent with the above summary of basic data. Higher blood counts of neutrophils, platelets, and monocytes and lower counts of lymphocytes have been shown to be associated with poor outcomes in gliomas [2,12,22]. Furthermore, higher NLR and PLR, and lower LMR are associated with shorter OS, PFS, a poorer outcome, and a higher grade of glioma [2,23,24].

The results of the current study are very well fit with the reported data in the literature. Overall, across the whole group, we showed that neutrophil and lymphocyte counts are associated with platelet counts, and, more importantly, neutrophil and monocyte counts correlated with mitosis and Ki-67 LI, indicators of cell proliferation, including tumor cells [8,25]. Likewise, NLR and LMR positively and *negatively* correlated with mitosis and Ki-67 LI, respectively. IDH1 mutation correlated with 1p19q codeletion and markers of good prognosis in gliomas [26,27]. Notably, IDH1 mutation *was negatively correlated with LMR, consistent with the literature, which indicates* that LMR is a poor prognostic marker in gliomas [24]. Estimated correlations between favorable and poor prognostic signs in our study suggest that preoperative systemic inflammatory markers may be indicated as potential prognostic factors in gliomas in the future.

There are a limited number of studies showing gender differences related to preoperative systemic inflammatory markers and selected poor pathological indices. Epidemiological studies have shown that the incidence of gliomas, including GBM, is consistently high in males across all age groups, and that females with HGGs have longer survival than males [28,29]. Higher PLR and NLR were observed in males, and some suggested that higher NLR correlated with glioma grade in males rather than in females. Our results showed higher levels of poor prognostic factors, including higher neutrophil and monocyte counts, NLR, mitosis, and Ki-67 LI, but lower LMR in males (Table 1). Males had significantly higher mitotic counts and Ki-67 LI, supporting the notion that gliomas are more common and more aggressive in males.

There are no reported data on whether side preference affects the parameters studied here, and our results suggest that side preferences do not affect these parameters, as expected (Table 2).

In the present study, significant differences in poor prognostic factors, including high neutrophil and low lymphocyte counts, NLR, mitosis, Ki-67 LI, and low LMR, were identified in patients with multilobar gliomas. Especially, very high mitotic activity and Ki-67 LI were present in multilobar gliomas, although the number of multilobar gliomas was significantly lower than that of lobar gliomas (Table 3). There is insufficient data in the literature, and only a limited number of studies have provided contradictory results. Some found no correlation between preoperative systemic inflammatory markers and focality [12], but others showed a larger tumor size associated with higher NLR [22]. We believe our results support the notion that multifocal involvement in glioma, especially in HGG, may lead to greater edema, which, in turn, drives a stronger inflammatory response. Furthermore, edema has been shown to be a poor prognostic factor in glioma [30], and, as expected, the current study found that poor prognostic factors are more common in multilobar gliomas.

Our results revealed that neutrophil-dependent inflammatory response increased while lymphocyte-mediated antitumor activity decreased. Higher neutrophil and monocyte counts, higher NLR, higher mitotic activity, higher Ki-67 LI, and lower LMR were common in our HGG and GBM groups (Tables 4 and 5). Although PLR was higher in HGG and GBM groups, the difference was not significant. We think that if we were able to include more patients, PLR would show significantly higher levels. Almost all metaanalyses demonstrated that the patients with a higher NLR had a worse prognosis, shorter OS and/or PFS, and were associated with a higher grade of gliomas [2,3]. Regarding PLR, the literature yields conflicting results. Some studies found that high PLR was associated with poor prognosis [10,18], whereas others found that PLR was not associated with poor prognosis in GBM [2]. An interesting finding across current and previous studies is that low LMR has been consistently reported in HGG and/or GBM [14,10,15,18]. Thus, it is worth mentioning in detail, and we think that future prospective studies will elucidate the importance of LMR, and a standard cut-off value needs to be deliberated for prognosis in gliomas. Low LMR results from decreased lymphocyte count and/or increased monocyte count. Monocytes, by secreting vascular endothelial growth factor and tumor necrosis factor-alpha, promote angiogenesis and tumor progression [20]. This explains why low LMR is associated with glioma grade and poor prognosis. Low LMR was negatively associated with NLR, PLR, mitotic activity, Ki-67, and IDH1 mutation in the present series, suggesting this marker could be a prognostic marker in gliomas.

A high level of mitosis is a poor prognostic sign, and the number increases in high-grade tumors [25]. It is not unexpected to find high Ki-67 LI in gliomas with high mitotic activity. In this context, our results fit well with the literature [10] (Table 6). It has been demonstrated that Ki-67 is a sensitive pathological marker for proliferating tumor cells and correlates with glioma grade [10,31]. Furthermore, we found that poor prognostic markers, including high neutrophil and/or monocyte counts, NLR, Ki-67 LI, and low LMR, were associated with gliomas with high mitotic activity. The presence of positive and negative correlations between NLR and mitosis, and between LMR and mitosis, suggested a biological link between systemic inflammation and glioma pathology. We, like others, underline that the results, including ours, need to be established by future prospective studies with a larger patient cohort.

IDH1 mutation and 1p/19q codeletion are well-known favorable prognostic factors in gliomas [7,26]. IDH1 mutations downregulate hypoxia-inducible factor (HIF) expression, a driving force of tumorigenesis [32]. It is accepted that HIF downregulation suggests that gliomas have lower levels of inflammation, which attracts fewer systemic neutrophils into the glioma microenvironment [7]. Studies have shown that in IDH1-mutant gliomas, NLR was significantly lower than in nonmutant gliomas and was an independent predictor of better survival [7]. Furthermore, IDH1-mutant GBMs have been noted to appear less invasive on magnetic resonance imaging than IDH1-nonmutant GBMs [33]. Codeletion of 1p/19q is associated with a good response to alkylating agent chemotherapy and is a favorable prognostic factor [34]. It has been demonstrated that 1p/19q codeletion is associated with longer survival and longer time to malignant transformation in diffuse LGG [26,34]. Even codeleted gliomas have significantly longer survival compared with undeleted gliomas [26]. Our results further supported that IDH-1-mutant and 1p/19q codeleted gliomas have less chronic inflammation. Unfavorable prognostic factors, including significantly higher mitotic activity and Ki-67 LI, were noted in IDH1-nonmutant and 1p/19q nondeleted gliomas. Moreover, low LMR and high NLR were found in IDH1-nonmutant gliomas, although some reported the opposite findings [7]. None of the unfavorable prognostic inflammatory markers showed a significant difference between 1p/19q codeleted and nondeleted gliomas, and we believe the main reason was the small number of gliomas; 1p/19q codeletion was evaluated (Tables 7 and 8). Taken together, our results support the idea that, in gliomas with IDH1 mutation and 1p/19q codeletion, there is lower chronic inflammation, which may partly explain the favorable prognosis of these tumors.

As expected, mitotic activity and Ki-67 LI showed a steady increase from grade I to grade IV (Table 9), with the highest values observed in grade-IV gliomas. Furthermore, the highest neutrophil counts and NLR, and the lowest LMR in grade-IV gliomas have caught our attention. Unfavorable prognostic markers, neutrophil count, and NLR were significantly higher, and LMR was significantly lower in grade-IV gliomas. Similarly, mitotic activity and Ki-67 LI were significantly higher in grade IV as well. Our results are consistent with the current literature [10,18,23] and suggest a biological link between unfavorable pathological markers, such as mitotic count and Ki-67, and preoperative systemic inflammatory markers, such as NLR and LMR. We underline that preoperative systemic inflammatory markers, especially NLR and LMR, may reflect the microenvironmental inflammatory process around a glioma and can serve as prognostic indices and predictors of grade. Future prospective studies should focus on NLR and LMR and determine a cut-off value, which may guide researchers in developing novel anti-inflammatory therapies and clinicians in predicting better survival.

The authors of the current report are aware of some limitations. 1) Our study is retrospective, and some unavoidable biases may exist. 2) The patients were enrolled from a single institution and a single surgeon. 3) We used the WHO classification of gliomas, released in 2016, and novel markers have only been incorporated recently in the pathological reports. 4) Depending on the inclusion criteria, a small sample was evaluated. 5) Tumor residual volume and extent of resection were not evaluated as the data were not available for all patients.

## 5. Conclusion

Our study demonstrated that poor prognostic preoperative hemogram parameters (higher levels of neutrophil and monocyte counts) and poor prognostic preoperative systemic inflammatory markers (higher NLR and lower LMR) are correlated with poor pathological indices (higher mitosis and Ki-67 LI). Likewise, higher NLR and lower LMR were significantly higher in multilobar gliomas, HGGs, GBMs, gliomas with higher mitosis, nonmutant gliomas, and gliomas without 1p/19q codeletion. Furthermore, as the WHO grade increases, markers suggestive of poor prognosis increase. These results suggest a link between systemic inflammation and histopathology, and that higher NLR and/or lower LMR could be a novel prognostic marker. A prospective multicenter study with a larger patient population would be more convincing. Until such a study is published, the results of retrospective studies, including ours, should be evaluated with caution.

## Figures and Tables

**Table 1:** Statistical summary of comparison between the genders with respect to preoperative systemic inflammatory markers and pathological molecular indices.

Variable	Male (n = 75; 60%)	Female (n = 50; 40%)	P-value
Neutrophil	2.81 (1.79–4)	2.63 (1.74–3.47)	†0.33
Lymphocyte	1.92 ± 0.91	2.10 ± 2.83	*0.60
Platelet	234.40 ± 71.83	265.42 ± 62.19	* **0.01**
Monocyte	0.55 (0.50–0.70)	0.48 (0.40–0.60)	† **0.04**
NLR	2.81 (1.79–4)	2.63 (1.70–3.30)	†0.33
PLR	122.22 (92.50–176.24)	139.16 (108.31–180.67)	†0.23
LMR	3.5 ± 2.0	4.12 ± 1.42	*0.09
**Mitotic activity	4 (1–12)	1 (0–8.5)	† **0.006**
Ki-67 LI (%)	10 (4–30)	5 (2–20)	† **0.009**

LI: Labeling index; LMR: Lymphocyte to monocyte ratio; NLR: Neutrophil to lymphocyte ratio; PLR: Platelet to lymphocyte ratio

* Independent samples t-test and values were expressed as mean ± standard deviation

† Mann–Whitney U test and values were expressed as median and percentiles (25^th^ – 75^th^ percentiles)

** Mitotic activity: Number of mitoses/10 HPF (high-power field) and the mean were evaluated here

**Table 2:** Statistical summary of comparison between the sides with respect to preoperative systemic inflammatory markers and pathological molecular indices.

Variable	Left (n = 66; 52.8%)	Right (n = 59; 47.2%)	P-value
Neutrophil	2.91 (1.87–3.91)	2.20 (1.65–3.95)	†0.21
Lymphocyte	1.87 ± 0.76	2.06 ± 1.0	*0.23
Platelet	253.19 ± 65.52	239.66 ± 73.75	*0.27
Monocyte	0.50 (0.40–0.60)	0.50 (0.40–0.70)	†0.44
NLR	2.91 (1.84–3.92)	2.20 (1.64–3.83)	†0.21
PLR	143.30 (106.80–184.69)	81.12 (91.77–172.49)	†0.05
LMR	3.67 ± 1.46	3.91 ± 2.20	*0.47
**Mitotic activity	3 (0–10)	3 (1–12)	†0.34
Ki-67 LI (%)	5 (2.5–25)	8 (3–30)	†0.35

LI: Labeling index; LMR: Lymphocyte to monocyte ratio; NLR: Neutrophil to lymphocyte ratio; PLR: Platelet to lymphocyte ratio

* Independent samples t-test and values were expressed as mean ± standard deviation

† Mann–Whitney U test and values were expressed as median and percentiles (25^th^ – 75^th^ percentiles)

** Mitotic activity: Number of mitoses/10 HPF (high-power field) and the mean were evaluated here

**Table 3:** Statistical summary of comparison between the lobes involved (lobar vs. multilobar) with respect to preoperative systemic inflammatory markers and pathological molecular indices.

Variable	Lobar (n = 99, 79.2%)	Multilobar (n = 26; 20.8%)	P-value
Neutrophil	2.40 (1.69–3.41)	3.79 (2.22–7.50)	† **0.007**
Lymphocyte	2.06 ± 0.87	1.57 ± 0.81	* **0.01**
Platelet	254.16 ± 63.98	218.80 ± 83.27	*0.05
Monocyte	0.53 (0.40–0.67)	0.52 (0.40–0.75)	†0.95
NLR	2.40 (1.68–3.44)	3.79 (1.97–7.15)	† **0.007**
PLR	131.11 (97.37–167.66)	147.86 (102.24–201.62)	†0.16
LMR	3.98 ± 1.90	3.04 ± 1.38	* **0.02**
**Mitotic activity	2.40 (1–9)	9.33 (1.5–21)	† **0.02**
Ki-67 LI (%)	5.64 (3–23.75)	21.66 (3.5–35)	† **0.04**

LI: Labeling index; LMR: Lymphocyte to monocyte ratio; NLR: Neutrophil to lymphocyte ratio; PLR: Platelet to lymphocyte ratio

* Independent samples t-test and values were expressed as mean ± standard deviation

† Mann–Whitney U test and values were expressed as mean and percentiles (25^th^ – 75^th^ percentiles)

** Mitotic activity: Number of mitoses/10 HPF (high-power field) and the mean were evaluated here

**Table 4:** Statistical summary of comparison between the grade (LGG vs. HGG) with respect to preoperative systemic inflammatory markers and pathological molecular indices.

Variable	LGG (n = 67; 53.6%)	HGG (n = 58; 46.4%)	P-value
Neutrophil	4.18 (1.64–3.25)	5.86 (1.97–6.09)	† **0.003**
Lymphocyte	2.06 ± 0.97	1.84 ± 0.76	*0.18
Platelet	243.22 ± 66.44	250.94 ± 73.39	*0.53
Monocyte	0.49 (0.40–0.60)	0.57 (0.5–7)	† **0.01**
NLR	2.20 (1.64–3.25)	2.93 (1.97–6.09)	† **0.01**
PLR	122.22 (104–122.22)	140.70 (95.84 ± 206.16)	†0.13
LMR	4.22 ± 2.04	3.28 ± 1.45	* **0.004**
**Mitotic activity	0.73 (0–2)	10.87 (7–16)	† **0.00001**
Ki-67 LI	3.20 (2–5)	27.33 (15–40)	† **0.00001**

HGG: High-grade glioma; LGG: Low-grade glioma; LI: Labeling index; LMR: Lymphocyte to monocyte ratio; NLR: Neutrophil to lymphocyte ratio; PLR: Platelet to lymphocyte ratio

* Independent samples t-test and values were expressed as mean ± standard deviation

† Mann–Whitney U test and values were expressed as median and percentiles (25^th^ – 75^th^ percentiles)

** Mitotic activity: Number of mitoses/10 HPF (high-power field) and the mean were evaluated here

**Table 5:** Statistical summary of comparison between GBM and non-GBM with respect to preoperative systemic inflammatory markers and pathological molecular indices.

Variable	GBM (n = 38; 30.4%)	Non-GBM (n = 87; 69.6%)	P-value
Neutrophil	7.03 (5.22–10.92)	4.23 (3.50–5.50)	† **0.00001**
Lymphocyte	1.79 ± 0.89	2.03 ± 0.87	*0.16
Platelet	242.36 ± 62.94	248.74 ± 72.54	*0.63
Monocyte	0.59 (0.40–0.90)	0.51 (0.40–0.60)	†0.05
NLR	3.81 (2.24–8.54)	2.20 (1.64–3.21)	† **0.00001**
PLR	158 (94.37–213.11)	122.14 (101.89–161.66)	†0.08
LMR	3.16 ± 1.63	4.06 ± 1.87	* **0.01**
**Mitotic activity	12.85 (9–20.25)	1.25 (0–40)	† **0.00001**
Ki-67 LI (%)	33.33 (25–50)	3.90 (2–10)	† **0.00001**

GBM: Glioblastoma multiforme; LI: Labeling index; LMR: Lymphocyte to monocyte ratio; NLR: Neutrophil to lymphocyte ratio; PLR: Platelet to lymphocyte ratio

* Independent samples t-test and values were expressed as mean ± standard deviation

† Mann–Whitney U test and values were expressed as mean and percentiles (25^th^ – 75^th^ percentiles)

** Mitotic activity: Number of mitoses/10 HPF (high-power field) and the mean were evaluated here

**Table 6:** Statistical summary of comparison between the presence and absence of mitoses (/10 HPF) with respect to preoperative systemic inflammatory markers and pathological molecular indices.

Variable	Presence (n = 94; 75.2%)	Absence (n = 31; 24.8%)	P-value
Neutrophil	5.38 (3.6–8.7)	4.08 (3.65–5.09)	† **0.01**
Lymphocyte	1.94 ± 0.90	2.0 ± 0.83	*0.74
Platelet	247.08 ± 74.21	245.96 ± 54.18	*0.93
Monocyte	0.55 (0.50–0.70)	0.44 (0.32–0.57)	† **0.01**
NLR	2.84 (1.83–4.90)	1.94 (1.68–3.0)	† **0.03**
PLR	137.17 (95.56–176.76)	122.94 (105.44–161.48)	†0.73
LMR	3.61 ± 1.94	4.32 ± 1.40	*0.06
Ki-67 LI (%)	15.71 (5–30)	2.31 (2.0–3.0)	† **0.00001**

HPF: High power field; LI: Labeling index; LMR: Lymphocyte to monocyte ratio; NLR: Neutrophil to lymphocyte ratio; PLR: Platelet to lymphocyte ratio

* Independent samples t-test and values were expressed as mean ± standard deviation

† Mann–Whitney U test and values were expressed as median and percentiles (25^th^ – 75^th^ percentiles)

**Table 7:** Statistical summary of comparison between the presence and absence of IDH1 mutation with respect to preoperative systemic inflammatory markers and pathological molecular indices.

Variable	Mutant (n = 71; 56.8%)	Nonmutant (n = 47; 37.6%)	P-value
Neutrophil	4.56 (3.5–4.5)	5.76 (4.2–9.7)	†0.01
Lymphocyte	1.99 ± 0.85	1.84 ± 0.85	*0.34
Platelet	245.56 ± 73.63	248.38 ± 67.96	*0.83
Monocyte	0.49 (0.40–0.60)	0.57 (0.40–0.70)	†0.14
NLR	2.47 (1.68–3.44)	2.93 (1.84–6.80)	† **0.03**
PLR	126.87 (101.88–163.06)	140.95	†0.22
LMR	4.07 ± 2.06	3.36 ± 1.48	* **0.04**
**Mitotic activity	1.87 (0–5.25)	11.40 (4.5–16)	† **0.00001**
Ki-67 LI (%)	4.88 (3–10)	40 (3–10)	† **0.00001**

IDH: Isocitrate dehydrogenase; LI: Labeling index; LMR: Lymphocyte to monocyte ratio; NLR: Neutrophil to lymphocyte ratio; PLR: Platelet to lymphocyte ratio

* Independent samples t-test and values were expressed as mean ± standard deviation

† Mann–Whitney U test and values were expressed as median and percentiles (25^th^ – 75^th^ percentiles)

** Mitotic activity: Number of mitoses/10 HPF (high-power field) and the mean were evaluated here

**Table 8:** Statistical summary of comparison between the presence and absence of 1p/19q codeletion with respect to preoperative systemic inflammatory markers and pathological molecular indices.

Variable	1p/19q + (n = 26; 20.8)	1p/19q – (n = 24; 19.2)	P-value
Neutrophil	4 (3.15–5.70)	2.40 (3.65–9.15)	†0.07
Lymphocyte	1.73 ± 0.68	1.98 ± 1.26	*0.38
Platelet	222.76 ± 77.35	257.50 ± 66.28	*0.09
Monocyte	0.47 (0.40–0.60)	0.47 (0.30–0.60)	†0.85
NLR	2.47 (1.65–3.95)	2.92 (2.20–5.18)	†0.28
PLR	125.27 (106.08–165–83)	147.74 (111.78–184.16)	†0.39
LMR	3.73 ± 1.45	4.24 ± 2.92	*0.42
**Mitotic activity	0.94 (0–6)	3 (2–9.75)	† **0.02**
Ki-67 LI (%)	4 (2.5–9)	6.66 (5–26.25)	† **0.04**

LI: Labeling index; LMR: Lymphocyte to monocyte ratio; NLR: Neutrophil to lymphocyte ratio; PLR: Platelet to lymphocyte ratio

* Independent samples t-test and values were expressed as mean ± standard deviation

† Mann–Whitney U test and values were expressed as median and percentiles (25^th^ – 75^th^ percentiles)

** Mitotic activity: Number of mitoses/10 HPF (high-power field) and the mean were evaluated here

**Table 9:** Statistical summary of comparison between the WHO glioma grades with respect to preoperative systemic inflammatory markers and pathological molecular indices.

Variable	WHO-I (n=13; 10.4%)	WHO-II (n = 53; 42.4%)	WHO-III (n = 21; 16.8%)	WHO-IV (n = 38; 30.4%)	P-value
Neutrophil	4 (3.40–4.30)	4.50 (3.50–6)	4.40 (3.75–5.17)	7.03 (5.22–10.92)	† **0.00001**
Lymphocyte	2.30 ± 1.0	1.98 ± 0.96	1.99 ± 0.44	1.79 ± 0.89	*0.34
Platelet	272.07 ± 48.44	237.11 ± 69.02	263.66 ± 88.80	242.36 ± 62.94	*0.27
Monocyte	0.60 (0.40–1)	0.50 (0.40–0.60)	0.55 (0.50–0.67)	0.59 (0.40–0.90)	† **0.02**
NLR	1.79 (1.51–2.56)	2.66 (1.69–3.75)	2.16 (1.57–2.93)	3.81 (2.24–8.54)	† **0.00001**
PLR	122.94 (120.45 ± 195)	122.22 (102.16 ± 161.48)	114.78 (96.45 ± 185.21)	158 (94.37 ± 213.11)	†0.38
LMR	3.84 ± 1.12	4.29 ± 2.21	3.60 ± 1.09	3.16 ± 1.63	* **0.03**
**MA	0.33 (0–1)	0.83 (0–2)	7.80 (4–10)	12.85 (9–20.25)	† **0.00001**
Ki-67 LI (%)	1.27 (1–2)	3.72 (2.25–5)	14.16 (5–23.75)	33.33 (25–50)	† **0.00001**

LI: Labeling index; LMR: Lymphocyte to monocyte ratio; MA: Mitotic activity; NLR: Neutrophil to lymphocyte ratio; PLR: Platelet to lymphocyte ratio; WHO: World Health Organization

* One-way ANOVA with post hoc Hochberg’s GT2 test, and values were expressed as mean ± standard deviation

† Kruskal–Wallis test and values were expressed as median and percentiles (25^th^ – 75^th^ percentiles)

** Mitotic activity: Number of mitoses/10 HPF (high-power field) and the mean were evaluated here
